# Medical News and Miscellany

**Published:** 1887-12

**Authors:** 


"DOC." W. M. YANDELL, so well known in Texas as a live newspaper man, and late President of the State Press Association, has just matriculated at Gross Medical College, Denver, Colorado, and will take the degaee "M. D." this spring.

Dr. Yandell is a brother of Prof. David W. Yandell, and of the late Prof· Lunsford P. Yandell, jr., of Louisville, and a son of the very distinguished Lunsford Yandell, sr., who was one of the founders of the University of Louisville, contemporary with the great Gross. This may account partly for the selection of the Gross Medical College away out in Denver. Dr. Yandell was lately quarantine officer at El Paso, under State appointment. He began the study of medicine in Memphis before the war, with his brother Dr. Lunsford P. Yandell, who was, at that time, in that college; attended a course at the Tulane University, in N. O., since the war, and now proposes to finish at the Gross, in Colorado. We congratulate the doctor, and commend the Gross Medical College to other gentlemen seeking to finish (or begin) a medical education.

---

DR. W. M. BURGER, late of Bartlett, Williamson county, Texas, has been appointed by the U. S. Government physician to the Tongue River Agency of Cheyenne Indians in Montana, and is now located at Tongue River. We are sorry to lose the doctor from Texas, but he directs the JOURNAL to be continued, and mailed to Tongue River.

Dr. J. L. Felder, of Leasburg, was incorrectly marked "deceased" on the roll of members of the T. S. M. S., in the Transactions, of 1886. As the doctor did not contradict it, the incoming secretary quite naturally dropped the name from the roll in the revision o the Transactions of 1887.

The circumstances are as follows: Dr. Felder attended the Dallas meeting in 1886, and served on the judicial committee, (and subscribed for this Journal). Shortly after the meeting Dr. Burt, the secretary, got news of the death of *Dr. Felder, at Leesburg.* But it transpires that it was Dr. Felder's son, Jno. L. Felder, jr., who resided at Pittsburg. He died at his father's house in Leesburg, and hence the confusion of names. We take pleasure in restoring Dr. Felder to membership in the T. S. M. A., and his name to the roll.

Apropos: Dr. Felder has removed from Leesburg to Cleburne, Texas. He has sent us a paper, giving his views as to the true cause of malarial fever, etc. This paper will be published in the next issue of the Journal, together with a description of the fungus, or spores—specimens of which Dr. Felder has sent us—as it appears under the microscope. The examination and description are by that able microscopist, Dr. Rudolph Menger, of San Antonio, and the description constituted part of the paper sent to be read at the late meeting of the Austin District Association, but which was, unfortunately, crowded out by the large number of papers presented by members in attendance. At an early day we will give an account of Dr. Menger's wonderful and valuable collection of photos and micro-photos of the "animals and insects of Texas." a monument to his zeal and patience in the cause of science.

---

### Personal.

Our esteemed friend and subscriber, Dr. Frank A. Maxwell, late assistant physician State Lunatic Asylum, Austin, has been tendered and has accepted and entered upon the duties of the position of Demonstrator of Anatomy in the Medical department of the University of Nashville and Vanderbilt University, Nashville, Tennessee. We congratulate the faculty on the acquisition of so able a man in this important office, and also extend our congratulations to our young friend on an opening which will doubtless call into early exercise and development his talents, and "lead to fortune and to fame." The newspapers married the doctor last month, but we can assure the "managing mammas" and the eligible young ladies of Nashville that this was unauthorized, not founded in fact, and was, altogether, too previous; but, Dr. Tom. O., his brother, (oh! Tom!) "stepped off" at Caldwell, lately. See notice.

---

PHYSICIAN'S MUTUAL BENEFIT ASSOCIATION OF TEXAS.

This organization is chartered, and is exempt from taxation. No duties, no meetings to attend, total expenses $1.00 a year, dues, and on the death of a member $1.00 to a fund for the widow. Every physician should *esteem it a privilege* to give a deceased brother's widow a dollar. It is often a godsend. Dr. S. F. Starley, at Tyler, Dr. W. T. Burts, at Fort Worth, and Dr. J. H. Bowers, at Columbus, will receive applications for membership. All members are authorized to do so, and we hope every brother will exert himself to get others to join. The present membership is 169; total enrollment, 211; total forfeitures, 38; total deaths in *four years*, 4!

A meeting of members will be held at Galveston during the session of the State Medical Association in April next.

The assessment for Mrs. Kilpatrick is not all paid yet. $100 has been sent her to date; the balance will be paid by January 1, when the time expires, and her receipt will be published in the January number.

---

IMPORTANT TO MEDICAL COLLEGES AND OTHER ADVERTISERS;: *The Dean of the St. Louis Post Graduate Medical College* writes, in renewing the announcement of that school: "We have so many students from your part of the country that we cannot afford to stop advertising." And later, "Yours is the *only journal* in which we continue to advertise; as it *certainly seems to have done us the most good.*" Strong language, but fact. Send for rates, which, in proportion to circulation, are most reasonable. Our list of advertisers, thirty-five pages, and all a 1, speaks well for the JOURNAL as a *Texas advertiser*.

---

DR. J. T. MUSICK, of Pittsburg, is in New York, attending the Polyclinic, and visiting the great hospitals.

COCAINE IN GOUT.

Dr. Edmund Goldmann, of San Jose, California, writes us :

"I recently had an opportunity of treating a severe case of gout in which there was extensive swelling of the big toe, with cocaine, locally. I used a six per cent. solution, with the most gratifying results ;—the cyanotic appearance being diminished in a few hours, and the excruciating pain subsided in twenty minutes, almost entirely. I have seen no published account of a similar case so treated. Perhaps you may have read of some such ; if not, you can speak of this one for the benefit of your many readers.

The solution of cocaine was applied by means of soft C. H. pencil, (about every five minutes), on the painful member,—until pain was relieved. Patient could not bear the slightest pressure or weight on the part, else I should have applied cotton pledgets saturated with the solution, in preference to painting with the C. H. pencil."

It does look as if, in cocaine, we have found the greatest boon to suffering man ; its uses are innumerable.

---

U. S. PENSION SURGEON.

We are gratified to learn that our greatly esteemed friend, subscriber and correspondent, Dr. O. Eastland, of Wichita Falls, has been appointed Examining Surgeon for U. S. Pensions.—"Uncle Sam" will have a vigilant watch kept on his treasury in North Texas, we'll warrant. The Pension Bureau are to be congratulated on so judicious a selection.

---

DR. W. H. WILKES : We have received information that Dr. Wilkes, who left Waco about a year ago, and accepted a chair in the Kansas City Medical College, has resigned the position and has returned to his old home. We welcome the Doctor back to Texas.

---

THE NORTH TEXAS MEDICAL ASSOCIATION — Dr. J. M. Fort, President ; Dr. G. R. Clayton, Secretary, has just closed a large, interesting and very instructive session at Gainesville. We are promised a full report of proceedings by the courteous Secretary, for the January Number of the JOURNAL. The interest in Medical organizations in Texas is ever increasing.

PERSONAL.—Our friend and subscriber, Dr. F. E. Yoakum, of Greenville, Texas, the able and popular President of the famous *East Line Medical Society*, is at present in Chicago, where he has entered the Illinois Eye and Ear Infirmary for a special course of instruction in ophthalmology and otology. He is also taking a course at the *Rush Medical College*; and at the College of Physicians and Surgeons a course on Chemistry and Microscopy. For some years Dr. Yoakum has limited his practice to the diseases of the Eye, Ear, Nose and Throat, and is well known throughout Texas as a skillful practitioner in those branches. Dr. Yoakum will contribute clinical reports for *this Journal* during his sojourn in the great medical center of the West.

